# Complete protection for mice conferred by a DNA vaccine based on the Japanese encephalitis virus P3 strain used to prepare the inactivated vaccine in China

**DOI:** 10.1186/s12985-020-01400-3

**Published:** 2020-08-24

**Authors:** Xiaoyan Zheng, Xiaozheng Yu, Yan Wang, Lance Turtle, Min Cui, Ran Wang, Chenghong Yin

**Affiliations:** 1 Beijing Institute of Tropical Medicine, Beijing Friendship Hospital, Capital Medical University, Beijing, 100050 China; 2 Beijing Key Laboratory for Research on Prevention and Treatment of Tropical Diseases, Beijing, 100050 China; 3 Department of Gastroenterology, Beijing Children’s Hospital, Capital Medical University, Beijing, 100045 China; 4 Outpatient Department, Beijing Friendship Hospital, Capital Medical University, Beijing, 100050 China; 5 NIHR Health Protection Research Unit for Emerging and Zoonotic Infections, University of Liverpool, Liverpool, L69 7BE UK; 6 Tropical and Infectious Disease Unit, Royal Liverpool University Hospital (member of Liverpool Health Partners), Liverpool, L7 8XP UK; 7 State Key Laboratory of Agricultural Microbiology, College of Veterinary Medicine, Huazhong Agricultural University, Wuhan, 430070 Hubei China; 8 Beijing Key Laboratory of Pediatric Respiratory Infection diseases, Key Laboratory of Major Diseases in Children, Ministry of Education, National Clinical Research Center for Respiratory Diseases, Research Unit of Critical Infection in Children, Chinese Academy of Medical Sciences, 2019RU016, Laboratory of Infection and Virology, Beijing Pediatric Research Institute, Beijing Children’s Hospital, Capital Medical University, National Center for Children’s Health, Beijing, 100045 China; 9 Department of Internal Medicine, Beijing Obstetrics and Gynecology Hospital, Capital Medical University, Beijing, 100026 China

**Keywords:** Japanese encephalitis virus, P3 strain, DNA vaccine, prM/E, Protection

## Abstract

**Background:**

The incidence of Japanese encephalitis (JE) has been dramatically reduced in China after sufficient vaccine coverage. The live-attenuated Japanese encephalitis virus (JEV) vaccine SA14–14-2 is believed to have strongly contribute to this decrease. Another vaccine that seems to have decreased in importance is an inactivated vaccine based on the JEV P3 strain, which is considered to be modifiable, such as being transformed into a DNA vaccine to improve its immunogenicity.

**Methods:**

In this study, the protective efficacy induced by the Japanese encephalitis DNA vaccine candidate pV-JP3ME encoding the premembrane (prM) and envelope (E) proteins of the P3 strain was assessed in BALB/c mice. The *prM/E* genes of the JEV P3 strain were subcloned into the vector pVAX1 (pV) to construct pV-JP3ME.

**Results:**

The plasmid DNA was immunized into BALB/c mice, and high titers of IgG antibody and neutralizing antibody (nAb) against JEV were detected. The key cytokines in splenocytes were secreted upon stimulation with JEV antigens. Finally, complete protective efficacy was generated after challenge with the JEV P3 strain in the mice.

**Conclusions:**

The DNA vaccine pV-JP3ME based on the JEV P3 strain in this study can induce specific humoral immune and cytokine responses and provide complete protection against JEV in mice.

## Background

Japanese encephalitis (JE) virus (JEV) is a single positive-strand RNA virus belonging to the family *Flaviviridae* genus *Flavivirus* [1]. JEV infection can cause severe encephalitis, neurological sequelae, and even death in children and adults [2]. JEV was first isolated in China in 1940 and is transmitted mainly by *Culex* mosquito bites, with swine and wintering waterfowl as amplifying hosts [3]. By the end of the 1980s, JEV infection had constantly been a serious threat to the health of many Asian children, and nearly half of all JE cases have occurred in China. Since the development of two types of vaccinations in China, an inactivated vaccine (strain P3) and the live attenuated vaccine strain SA14–14-2 [4, 5], the incidence of JE has decreased from 20.92 cases/100,000 individuals in 1971 to 0.12 cases/100,000 individuals in 2011 [6, 7]. China is still using the two vaccines mentioned above, and live-attenuated vaccines were included in the national Expanded Programme on Immunization (EPI) at the end of 2007 [8]. Given the more convenient schedule, reduced toxicity, and better immunogenicity of live-attenuated vaccines, SA14–14-2 has now replaced inactivated vaccines [9]. Populations in some Asian countries and regions, such as Japan, South Korea, Taiwan, and certain populations, including immunocompromised people and those concerned about live vaccination, are still using inactivated vaccines [10]. The wild-type P3 strain has strong virulence and contains many key epitopes of immunogenicity, which can be improved by biological modification. In this study, by combining the biological characteristics of the virus and the advantages of the DNA vaccine, the *prM/E* genes of the P3 strain were subcloned into the DNA vaccine vector pVAX1 (pV) to obtain the JEV DNA vaccine pV-JP3ME. The comprehensive immune response and protection induced by this vaccine were evaluated, and this study will provide important data for its further application.

## Materials and methods

### Virus, cells, plasmids, and animals

JEV (strain P3) was stored at − 80 °C. It was used as the coating antigen and stimulus for in vitro experiments and used for challenge experiments.

Vero cells were used for plasmid transfection and plaque assays to detect viral titers, and the plaque reduction neutralization test (PRNT) was used to detect the nAb titers. C6/36 cells are used for virus proliferation assays.

The pV-JP3ME plasmid was constructed by introducing the *BamH*I enzyme digestion site, Kozak sequence and signal sequence upstream of the *prM/E* sequence of the P3 strain and introducing the *Xho*I digestion site downstream into the eukaryotic expression vector pV.

Specific pathogen-free 6- to 8-week-old female BALB/c mice were used for immunity, sera and splenocyte collection and challenge tests. The results presented are from a single experiment or are from three independent experiments.

### Reagents and instruments

The restriction enzymes *BamH*I and *Xho*I, eukaryotic expression vector pV, nuclear staining agent 4′,6-diamidino-2-phenylindole (DAPI), and transfection reagent Lipofectamine 3000 were purchased from Thermo Scientific (USA). Minimal essential medium (MEM) and RPMI-1640 medium were purchased from Gibco (USA). Methylcellulose was purchased from Sigma (USA). Goat anti-mouse fluorescein isothiocyanate (FITC)-IgG antibody was purchased from Beijing TransGen Biotech (China). Goat anti-mouse horseradish peroxidase (HRP)-IgG antibody was purchased from Abcam (USA). Tetramethylbenzidine (TMB) substrate color solution was purchased from MabTech Company (USA). The enzyme-linked immunospot (ELISPOT) kit, streptavidin and AEC color development kit were purchased from BD Company (USA). The gene introduction instrument was purchased from Shanghai Teresa Corporation (China). The enzyme-linked immunosorbent assay (ELISA) plate reader and cell culture incubator were purchased from Thermo (USA). The ELISPOT plate reader was purchased from CTL (USA).

### Transfection and immunofluorescence experiments

pV-JP3ME or pV was transfected into Vero cells. After 5 h, the transfection plasmid/reagent mixture was discarded and replaced with complete culture medium. After 40 h, the medium was discarded, and the two groups of cells were simultaneously fixed. Then, the cells were incubated with JEV antiserum (1:1000) as the primary antibody and goat anti-mouse FITC-IgG as the secondary antibody. Observation of specific green fluorescence under a fluorescence microscope indicates that the plasmid was successfully transfected and expressed in mammalian cells in vitro. Fluorescence microscope imaging was performed at 200× magnification, and the microscope was from Nikon, Japan.

### Animal experiments

**Fig. 1:**
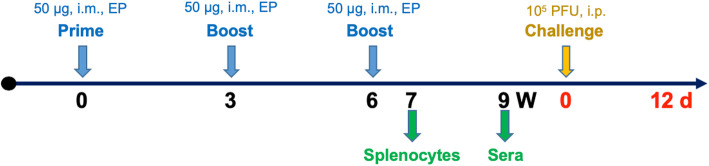
Mouse experimental workflow. Groups of mice were immunized by intramuscular electroporation with 50 μg of either the pV-JP3ME DNA vaccine or pV in each limb individually and were boosted twice at three-week intervals. Splenocytes were obtained 1 week after the final immunization, and sera were collected 3 weeks after the final immunization. Subsequently, the vaccinated mice were challenged with 1 × 10^5^ PFU of the JEV P3 strain. The body weight changes and the survival rates were observed for 12 consecutive days after the challenge

The mice were randomly divided into two groups. The vaccine group was vaccinated with the DNA vaccine pV-JP3ME, and the control group was vaccinated with the empty vector plasmid pV. Immunization was performed three times, and each immunization dose was 50 μg. Intramuscular injection (i.m.) with electroporation (EP) was used. One and 3 weeks after the last immunization, the splenocytes and sera of the two groups of mice were collected, respectively. The next day, mice in the vaccine and the control group were challenged with JEV. The body weight changes and the survival rate were measured daily after the challenge, and the observation continued for 12 consecutive days. The animal experiment schedule is shown in Fig. 1.

### Plaque assay

Vero cells were cultured in 24-well plates, and the cell density was more than 95% before virus infection. The serially diluted virus was serially diluted 10-fold from the original solution (1:1) for a total of seven dilutions, namely, 1:1 to 1:10^6^. Two hundred microliters of the virus dilution was added to each well and incubated at 37 °C for 1 h. The plate was gently shaken once every 15 min. After the dilution was discarded, 5 mL of MEM medium containing 1.2% methylcellulose was added to each well. After 4 d of culture, the medium was discarded, and 1 mL of crystal violet staining solution was added to each well and stained for 30 min at room temperature. The number of plaques per well was counted, and the virus titer was calculated. The titer of the virus solution was repeatedly measured three times, and an average value was taken and is expressed as the plaque-forming unit (PFU)/mL.

### ELISA

Heat-inactivated JEV was coated in a 96-well plate at 10^5^ PFU per well at 4 °C overnight. The coating antigen was discarded and blocked with 1% bovine serum albumin (BSA) at 37 °C for 2 h. The blocking solution was discarded. The sera of each group of mice were started from 1:100 and were serially diluted at a twofold ratio for a total of 12 dilutions, namely, from 1:100 to 1:204,800, and added to the wells in turn as a primary antibody at 4 °C overnight. The next day, the primary antibody was discarded. After the plate was washed five times, HRP-labeled goat anti-mouse IgG antibody (1:4000) was added as a secondary antibody. After incubation at 37 °C for 1 h, the plate was discarded. The substrate solution developed color for 20 min, and the reaction was stopped with H_2_SO_4_. We used 1/2 of the A_450_ nm value at the 1:100 dilution of the control group as the cut-off value, and the maximum dilution greater than this cut-off value is the serum IgG antibody titer.

### PRNT

Vero cells were cultured in 24-well plates as described previously. The serum was diluted from 1:10 and serially diluted at a 2-fold ratio. There are seven consecutive dilutions, that is, 1:10 to 1:640. Each dilution of serum was mixed with an equal volume of virus solution (containing 100 PFU). For incubation at 37 °C for 1 h, serum-free virus samples were set at 4 °C and 37 °C to exclude temperature factors and reference positive virus counts. Then, the serum/virus mixture was added to the wells in order and incubated at 37 °C for 1 h. During the period, the plate was shaken gently every 15 min. The subsequent step was the same as described in 2.5. The serum dilution corresponding to a 50% reduction in the number of plaques in the positive wells incubated at 37 °C was recorded as the PRNT_50_ value, which is the nAb titer.

### ELISPOT assay

IL-2 and IFN-γ capture antibodies diluted 1:200 were coated in a 96-well plate at 4 °C overnight. We discarded the coating solution and blocked with RPMI-1640 medium containing 10% FBS at room temperature for 2 h. Then, the splenocytes from the two groups were added to each well at 2 × 10^5^ cells per well, and 10^5^ PFU heat-inactivated JEV was added as a stimulus and cultured at 37 °C for 72 h. After the cultured splenocytes were discarded, IL-2 and IFN-γ detection antibodies were added, and then, the spot forming units (SFUs) were determined by adding streptavidin and AEC chromogenic solution.

### Statistical analysis

All experimental data were recorded using Excel 2016 software, statistical analysis was performed using SPSS 17.0 software (USA), the body weight change was analyzed by repeated analysis of multivariate analysis of variance, survival rates were compared using the log-rank test, and the differences between the groups were compared using one-way ANOVA. Quantitative data are expressed as the mean ± standard deviation. *P* < 0.05 was considered statistically significant.

## Results

### The target protein prM/E was successfully expressed in eukaryotic cells

**Fig. 2:**
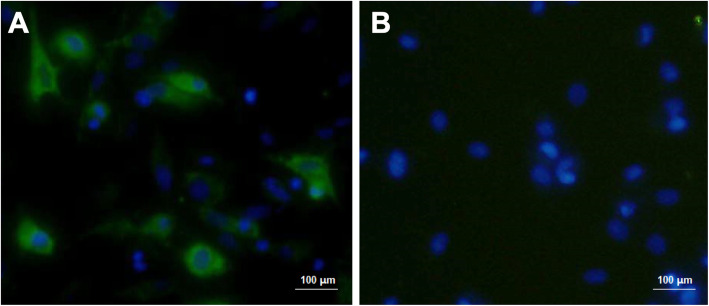
Representative images of immunofluorescence after Vero cells were transfected with plasmid DNA. After Vero cells were transfected with pV-JP3ME or pV, JEV antiserum was used as the primary antibody, and goat anti-mouse FITC-IgG was used as the secondary antibody for staining. The left image **a** shows specific green fluorescence, but the right image **b** does not (× 200)

As shown in Fig. 2, after Vero cells were transfected with pV-JP3ME, the expressed target protein could bind to the JEV antisera and showed specific green fluorescence, while the Vero cells transfected with the empty vector plasmid pV showed no specific fluorescence. These results indicated that the target protein prM/E can successfully be expressed in eukaryotic cells and has reactogenicity, which can be used for subsequent experimental research.

### Vaccination of mice with pV-JP3ME induces high levels of JEV-specific IgG antibodies in immune sera

**Fig. 3:**
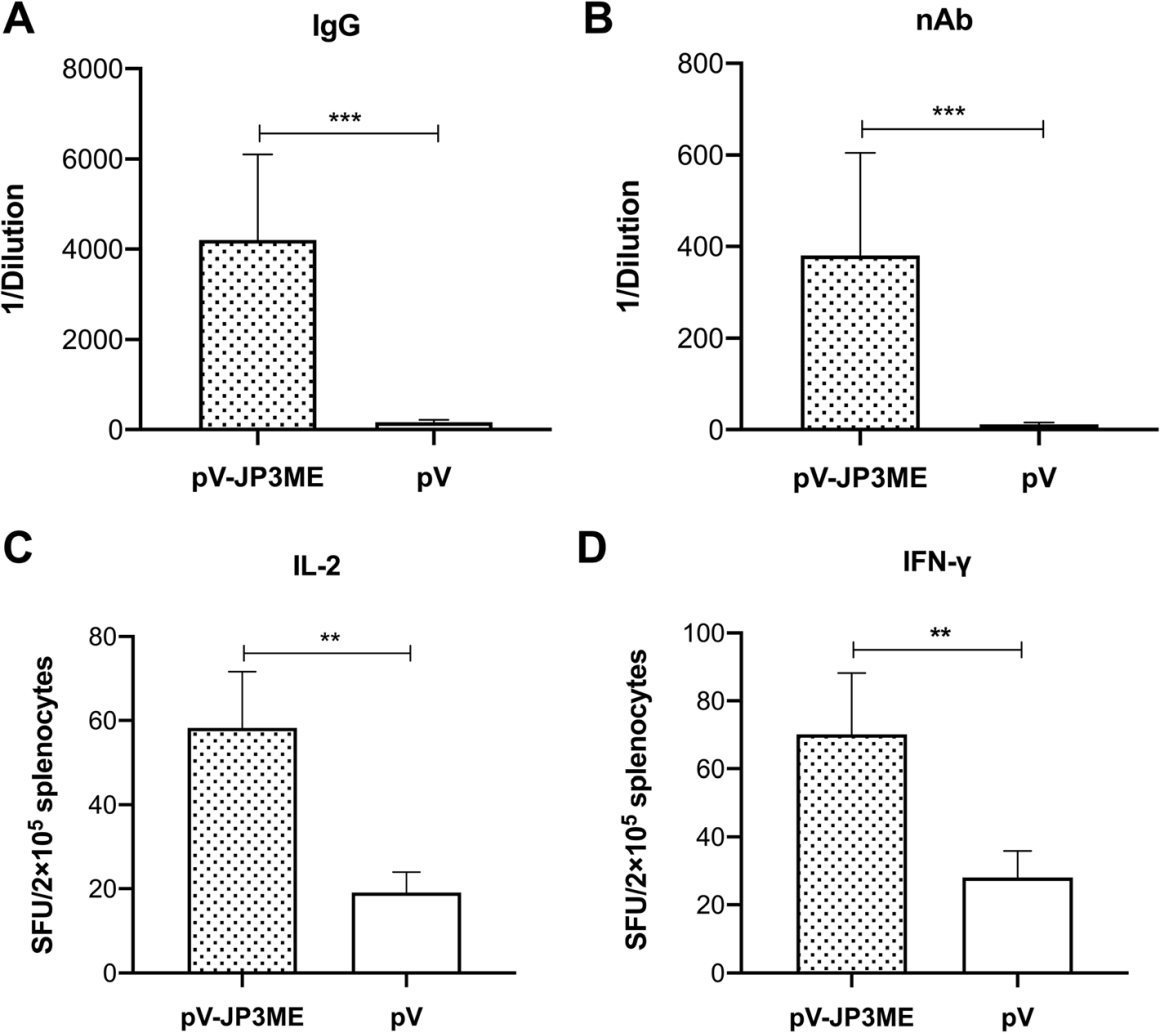
Specific humoral immune response and cytokine secretion produced by mice after immunization. In the third week after the mice were immunized three times with pV-JP3ME or pV, **a** IgG antibodies and **b** nAbs were produced with higher titers in the vaccine group than in the control group. One week after immunization, the mouse splenocytes produced high levels of **c** IL-2 and **d** IFN-γ upon JEV antigen stimulation. **P < 0.01, ***P < 0.001, *n* = 8 per group

As shown in Fig. 3a, 3 weeks after the last immunization, the sera of the vaccine group and the control group were collected, and the titer of the anti-JEV-specific IgG antibodies was measured by ELISAs. Higher IgG antibodies against JEV were detected in the sera of mice in the vaccine group than in the control group, with a titer of 1:4200 compared with 1:163 in the control group (*P* < 0.001). There is a significant difference between the groups, as shown in Fig. 3a, indicating that after immunization with the JEV DNA vaccine pV-JP3ME, mice can induce high levels of IgG antibodies against JEV.

### Robust neutralizing activity was found in the immune sera

The titer of anti-JEV-specific nAb was measured by PRNT. The results showed that the nAb titer against JEV in the sera of the vaccine group was 1:380 and that in the control group was 1:11 (*P* < 0.001). There was a significant difference between the groups, as shown in Fig. 3b, suggesting that after pV-JP3ME immunization, mouse sera had neutralizing activity against JEV.

### Inflammatory cytokines were produced upon stimulation with the JEV antigen

One week after the last immunization, splenocytes of the vaccine group and the control group were obtained, and ELISPOT was used to determine the number of IL-2- and IFN-γ-spots forming cells per 10^5^ splenocytes upon stimulation with the JEV antigen. The results showed that the spots of IL-2 and IFN-γ were significantly greater in the splenocytes of the vaccine group than in those of the control group in vitro (*P* < 0.01), as shown in Fig. 3c and d. After the mice were immunized with pV-JP3ME, the splenocytes secreted higher levels of IL-2 and IFN-γ after stimulation with the JEV antigen, suggesting a better antiviral cytokine response.

### Vaccinated mice were fully resistant to lethal doses of JEV

**Fig. 4:**
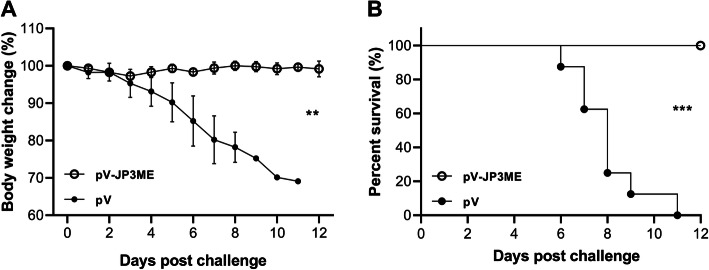
Protective effect generated by mice after immunization. In the third week after the mice were immunized with 3 pV-JP3ME or pV doses, the challenge test was performed. The mice in the vaccine group had no significant body weight changes within 12 days, while the control group showed a continual decline. Similarly, the mice in the vaccine group were completely protected, and the survival rate was up to 100%, while the mice in the control group all died. **P < 0.01, ***P < 0.001, *n* = 8 per group

To evaluate the protective efficacy of the JEV DNA vaccine pV-JP3ME in mice, we used 1 × 10^5^ PFU of JEV to intraperitoneally (i.p.) challenge the mice in the vaccine and the control group, and the changes in body weight and the survival rate were recorded and compared for 12 consecutive days. After the challenge with JEV, the body weight change of the vaccinated mice showed a steady trend during the observation period, and the differences between individuals were limited, while the body weight of the control mice continued to decline. A significant difference was shown between the two groups (*P* < 0.01, Fig. 4a). In terms of the survival rate, the mice in the vaccine group were fully protected, with a survival rate of 100% (8/8), while the mice in the control group began to die on the sixth day, and all died on the twelfth day (0/8). The difference between the groups was statistically significant (*P* < 0.001, Fig. 4b).

## Discussion

Currently, the JEV vaccine widely used in China is the live attenuated vaccine strain SA14–14-2. This vaccine has a good efficacy and, in addition to China, many Asian countries have approved it for use. Furthermore, another self-developed inactivated vaccine, the P3 strain, is still in use in China. The P3 strain used for special populations (based on concerns about live vaccines or unsafe vaccine incidents in society). However, the P3 inactivated vaccine rather than the SA14–14-2 vaccine is not included in the EPI; the former requires full payment by the patient, but it still has a solid market share as described above. In some Asian countries and regions, such as South Korea, Japan, and Taiwan, inactivated vaccines are customary and considered safe. Most of the inactivated vaccines used are the Nakayama strain [11], which originated from Japan and still predominates in the JEV vaccine market, although different types of live attenuated vaccines have been introduced into these regions. Inactivated vaccines do have some disadvantages that cannot be compared with live-attenuated vaccines, such as low immunogenicity, a significant Th2-type immune trend, and a requirement for multiple immunizations [12]. However, in terms of its safety, inactivated vaccines do not show virulence recovery and reproducibility, which still makes them highly desired in many JE-affected countries. The vaccinated population is mainly children around the age of 1 year, and safety cannot be ignored.

The SA14–14-2 live-attenuated vaccine requires only two injections, while the inactivated P3 vaccine requires four or more vaccinations to achieve sufficient protection. This phenomenon strongly limits the vaccine as the first choice for emergency vaccines for travelers. Even in JE-endemic countries such as China, this vaccine is not optimal in comparisons. It is difficult to develop a live attenuated vaccine based on the P3 strain, and even if it is successful, it is difficult to compete with the existing SA14–14-2 strain because the latter has been certified and recommended by the World Health Organization. In comparison, DNA vaccines have been used in vaccine design in recent years to prevent and treat multiple pathogens and diseases, such as infectious diseases including dengue virus, human immunodeficiency virus, and malaria and tumors [13, 14]. DNA vaccines have been approved for veterinary use, but there are no approved vaccines for human use [15]. Due to the balanced immune response and the long-lasting effect induced by DNA vaccines, they have become a popular alternative.

Therefore, in this study, the most antigenic structural protein, prM/E, of the JEV strain P3 was used as a target to construct a DNA vaccine. First, we verified the success or failure of expressing the target antigen in the transfection experiments of mammalian cells in vitro. The vaccine expresses the target protein, which can stably bind to JEV-specific antibodies, suggesting that it has good reactivity. Regarding the route, time and dose of immunizations, we have already fully studied other flavivirus vaccines, such as dengue virus and Zika virus, and will not discuss them here.

After three immunizations with the vaccine, mice show high titer IgG antibodies and nAb in vivo. Furthermore, the splenocytes of the immunized mice produced high levels of the cytokines IL-2 and IFN-γ upon stimulation with JEV-specific antigens. IL-2 plays an important role in the maturation, proliferation, and activation of T cells. IFN-γ is one of the most important innate and acquired antiviral cytokines and can play an antiviral role in both innate and adaptive immunity [16]. We selected these two representative cytokines for testing, suggesting that the vaccine is immunogenic. Furthermore, in the challenge test in vivo, pV-JP3ME provided complete protection for the mice, resulting in resistance to the lethal dose of JEV, while the mice in the control group all died. The above results indicate that the prM/E protein of the P3 strain is sufficient as a target protein candidate of the JEV vaccine to induce effective immunoprotection [17].

In recent years, there have been many studies on SA14–14-2, and the results showed the scalability of the vaccine. Erra et al. prepared an inactivated vaccine by using the live attenuated vaccine SA14–14-2 strain [18], and Appaiahgari et al. replaced the prM/E of the yellow fever virus attenuated strain 17D with that of SA14–14-2 by using chimeric recombination [19]; they also obtained a good immune effect. One of the reasons for selecting the P3 strain in this study is that the virulence of the P3 strain is closer to that of the wild type strain than SA14–14-2, and it is presented in the form of a DNA vaccine to ensure that there is no possibility of virulence recovery. The expressed target protein is similar to the natural conformation of the original strain prM/E protein containing key epitopes that are not displayed in the attenuated strain. Second, P3 is an inactivated vaccine strain widely used in China, and recombinant construction can be used in combination with the P3 inactivated vaccine in heterogeneous immunization in subsequent studies. Previous studies have reported that a DNA vaccine constructed using a specific target protein can be used as the prime immunization, a protein component vaccine (subunit vaccine) is prepared with the same target protein is boosted, and the immune response is more robust than that of homologous immunization, namely, DNA alone or subunit immunization alone [20]. The level of the immune response induced by heterologous immunization is higher and more balanced than single immunization, and the protective effect is also improved. This strategy is expected to reduce the times of immunization compared with immunization with an inactivated vaccine alone, and theoretically, it can also improve the immunogenicity and long-term immune response induced by the vaccine [21, 22], which warrants further experiments.

## Conclusions

In summary, this study used the P3 strain to construct a JEV DNA vaccine candidate, evaluated its immunogenicity and protective effect in mice, and confirmed that the vaccine can induce JEV-specific humoral and cytokine responses and provide complete protection against JEV in mice. Our data will provide a basis for the subsequent promotion and use of the vaccine and lay the foundation for its combined use with inactivated vaccines of the same strain in a heterologous regimen.

### Availability of data and materials

All data and materials described in the manuscript are available.

## Data Availability

The datasets used during the current study are available from the corresponding author on reasonable request.

## Abbreviations

JE: Japanese encephalitis
JEV: Japanese encephalitis virus
prM: Premembrane
E: Envelope
pV: pVAX1
nAb: Neutralizing antibody
EPI: Expanded Programme on Immunization
PRNT: Plaque reduction neutralization test
DAPI: 4′,6-diamidino-2-phenylindole
MEM: Minimal essential medium
FITC: Fluorescein isothiocyanate
HRP: Horseradish peroxidase
ELISPOT: Enzyme-Linked ImmunoSpot
ELISA: Enzyme-linked immune-sorbent assay
